# Role of Increased Lipoprotein (a) in Retinal Vein Occlusion: A Systematic Review and Meta-analysis

**DOI:** 10.1055/s-0041-1732803

**Published:** 2021-07-06

**Authors:** Francesco Paciullo, David Giannandrea, Gianni Virgili, Carlo Cagini, Paolo Gresele

**Affiliations:** 1 Division of Internal and Cardiovascular Medicine, Department of Medicine and Surgery, University of Perugia, Perugia, Italy; 2 Division of Neurology and Stroke Unit, Department of Neurology, Gubbio and Città di Castello Hospital, Perugia, Italy; 3 Department of Neurosciences, Psychology, Drug Research and Child Health (NEUROFARBA), Ophthalmology Clinic, University of Firenze and AOU Careggi, Florence, Italy; 4 Section of Ophthalmology, Department of Medicine and Surgery, University of Perugia, Perugia, Italy

**Keywords:** cardiovascular risk, retinal vein occlusion, unusual site vein thrombosis

## Abstract

**Background** Increased lipoprotein (a) [Lp(a)] has been associated with enhanced risk of cardiovascular events and more recently with venous thromboembolism. However, there is inconclusive data on the association between enhanced Lp(a) and retinal vein occlusion (RVO). We aimed to assess the role of Lp(a) in RVO.

**Methods** We performed a systematic review and meta-analysis of the studies addressing the role of Lp(a) in RVO. A systematic literature search was performed to identify all published papers reporting Lp(a) levels. Main outcome measures consisted of Lp(a) levels in patients with (cases) or without (controls) RVO.

**Results** We included 13 studies for a total of 1,040 cases and 16,648 controls. Lp(a) levels above normal limits were associated with RVO (OR 2.38, 95% CI 1.7–3.34) and patients with RVO had higher Lp(a) levels than controls (weighted mean difference: 13.4 mg/dL, 95% CI 8.2–18.6).

**Conclusion** Increased Lp(a) levels associate with RVO and should be included among diagnostic and prognostic indexes for this unusual-site vein thrombosis. Therapeutic interventions aimed to lower Lp(a) should be tested in RVO patients.

## Introduction

Retinal vein occlusion (RVO) is due to the thrombotic obstruction of retinal veins. Affecting 16 million people worldwide, RVO is the second most common retinal disease after diabetic retinopathy and it may be associated with serious consequences such as neurovascular glaucoma, retinal detachment, and ultimately blindness. 1 Based on the site of vascular occlusion RVO is distinguished in central retinal vein occlusion (CRVO), located in the central retinal vein at the passage through the lamina cribrosa, branch retinal vein occlusion (BRVO), involving one of the branches of the central retinal vein at an arteriovenous crossing, and hemispheric retinal vein occlusion (HRVO), involving the venous return from approximatively one half of the retina. BRVO is four times more common than CRVO, while bilateral vein thrombosis is very rare. 1 BRVO usually manifests as a sudden painless decrease in vision or a visual field defect, while CRVO usually presents with sudden, unilateral, painless loss of vision. 2 Unlike other vein thromboses, thrombophilia does not seem to play a major role in RVO, a conclusion supported by a recent meta-analysis which questioned the role of thrombophilia in retinal arterial and venous occlusive disease. 3 On the other hand, several common cardiovascular risk factors, such as hypertension, diabetes, and hyperlipemia, were reported to be predisposing factors for RVO 4 and to enhance the risk of RVO recurrence. 2 4 These findings suggest that although RVO is a venous thrombosis it has more characteristics in common with atherosclerosis than with venous thromboembolism (VTE). 3 4 5 Accordingly, despite some evidence of efficacy, 6 anticoagulation is seldom administered to these patients and it is mostly reserved to younger subjects with acute occlusion, while cardiovascular risk factors control is the most widely used and strongly recommended measure to prevent RVO recurrence. 2 Because of these uncertainties, medical management of this frequent and disabling condition is still far from optimal and a step forward in the knowledge of RVO pathogenesis is strongly required to identify appropriate therapeutic targets.

Lipoprotein (a) [Lp (a)] is a complex lipoprotein involved in tissue repair and wound healing. 7 Lp(a) resembles structurally low density lipoproteins (LDLs) from which it differentiates for the presence of apolipoprotein (a) [(apo(a)], a complex glycoprotein covalently bound to apo(b) by a disulfide bond. 8 Due to its high affinity for glycosaminoglycans of the human arterial wall, even higher than that of LDL, Lp(a) easily accumulates in the intima of large and medium size arteries where it promotes monocyte and macrophage recruitment and activates a local inflammatory response favoring atheroma development and finally arterial thrombosis. 9 Moreover, its apo(a) moiety competes with plasminogen, with which it shares more than 80% structural homology, thus exerting an antifibrinolytic effect. 7 8 Finally, Lp(a) promotes platelet aggregation through mechanisms incompletely understood, induces the synthesis of plasminogen activator inhibitor, and depresses the synthesis of tissue factor pathway inhibitor in all enhancing blood coagulation. 7 8 10 Unfortunately, even if some therapies have been described to lower L(a) levels, no specific treatment is still available to manage hyper-lp(a)-lipoproteinemia. Mendelian randomization studies confirmed a wide, genetically determined interindividual variation of Lp(a) circulating levels and molecular dimensions. 9 Interestingly, the small dimension Lp(a) phenotype, the most proatherogenic, is usually associated with high plasma levels configuring a high CV risk profile. 11 12 Indeed, large population studies and meta-analyses indisputably showed that increased Lp(a) levels are associated with cardiovascular events. 12 In particular, a strong correlation between Lp(a) levels and myocardial infarction or stroke risk has been shown in several prospective and retrospective population studies. 13 14 15 16 17 18 19 On the contrary, the role of Lp(a) in VTE is less clear. 20 21 In fact, while a recent systematic review and meta-analysis including 14 studies for a total of 14,000 patients concluded that Lp(a) levels associate with increased risk of VTE, 20 a subsequent prospective study from the Kuopio Ischemic Heart Disease cohort in 2,180 men followed for a median period of 24.9 years resolved the opposite. 21 Therefore, the association between Lp(a) and VTE remains controversial.

Concerning RVO, several prospective and retrospective cohort studies have reported an association between elevated Lp(a) levels and retinal vessel occlusive disease. 22 23 24 25 26 27 28 29 30 31 32 33 34 However, due to the small number of patients enrolled in the individual studies, the exact role of Lp(a) in RVO risk remains uncertain. Aim of the present study was to carry out a systematic review and meta-analysis of the studies evaluating the association between Lp(a) levels and RVO incidence.

## Methods

This systematic review and meta-analysis was performed following the PRISMA guidelines ( www.prisma-statement.org ) and it has been submitted to the International Prospective Register of Systematic Reviews (PROSPERO) (ID: 196552). Search strategies, methods for study quality assessment, and statistical plan were established a priori as well as the inclusion criteria and outcomes.

### Search Strategy

We performed an electronic search through the Scopus, PubMed, and Google Scholar databases using the keywords “retinal vein occlusion” OR “RVO” OR “retinal vein thrombosis” and “lipoprotein (a)” OR “Lp(a),” without data or language restrictions, up to September 21, 2020. The titles, abstracts, and full text of all retrieved documents were carefully evaluated, and the reference list of all papers was examined to extract articles of potential interest and those reporting data on Lp(a) levels in RVO were included in the analyzed literature.

### Study Selection and Data Extraction

Study selection was independently made by two reviewers (F.P. and D.G.) and disagreements were solved through discussion and when required with the opinion of a third investigator (P.G.). All case–control studies on patients with CRVO or BRVO reporting Lp(a) plasma levels were considered eligible for analysis with no restrictions about gender or age. Gray literature, or evidence not published in commercial publications was included in the systematic review. Data on arterial retinal occlusion, when available were not included in the analysis. Case reports were not included.

### Statistical Analysis and Risk of Bias Assessment

A meta-analysis was carried to calculate the individual and pooled odds ratios (ORs) and their relative 95% confidence intervals (95% CI). The analysis was performed using Review Manager (Version 5.4). A random effect model was applied to evaluate the ORs of the association between high Lp(a) levels and RVO. *Z* -scores were used to test the overall effect with *p* < 0.05 for significance. Results were presented with 95% CI. *I* ^2^ statistic and the Chi-square Cochrane *Q* test were performed to evaluate statistical heterogeneity, which was considered significant when *p* < 0.1. Attributable risk fraction was calculated as [P(RR − 1)/P(RR–1) + 1], where *P* = prevalence of risk factor in the population and RR is the relative risk. 35 Lp(a) levels were expressed as mg/dL. Publication bias was graphically analyzed by funnel plot. When Lp(a) data were reported as medians or means, a weighted mean difference (WMD) was calculated, and sample means and standard deviations were estimated and data meta-analyzed. When Lp(a) levels were expressed as medians and IQR, means and SD were estimated as previously described. 36 37 In the study of Gumus et al, RVO Lp(a) mean levels and SD were obtained from the mean levels and SD of two groups (BRVO and CRVO). To avoid possible bias related to the variability of measures among studies, a random effect model was applied. Similarly, a random effect model was used to evaluate the association between Lp(a) plasma levels and RVO.

## Results

**Fig. 1:**
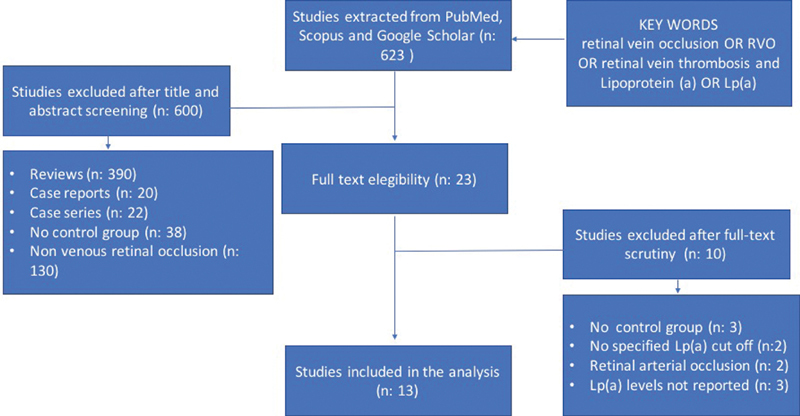
Search strategy and study selection.

**Fig. 2:**
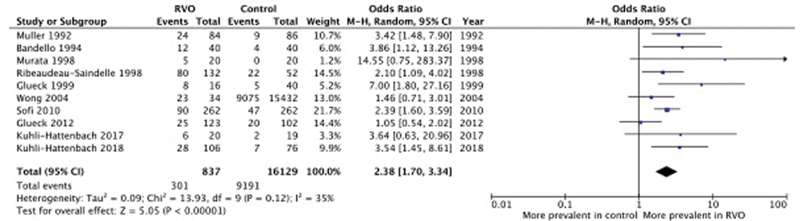
Prevalence of RVO in subjects with abnormal Lp(a) versus Lp(a) within normal range. Forest plot of the studies in which abnormal Lp(a) was defined by values above a prespecified upper normal limit. CI, confidence interval. Lp(a), lipoprotein (a); RVO, retinal vein occlusion.

**Fig. 3:**
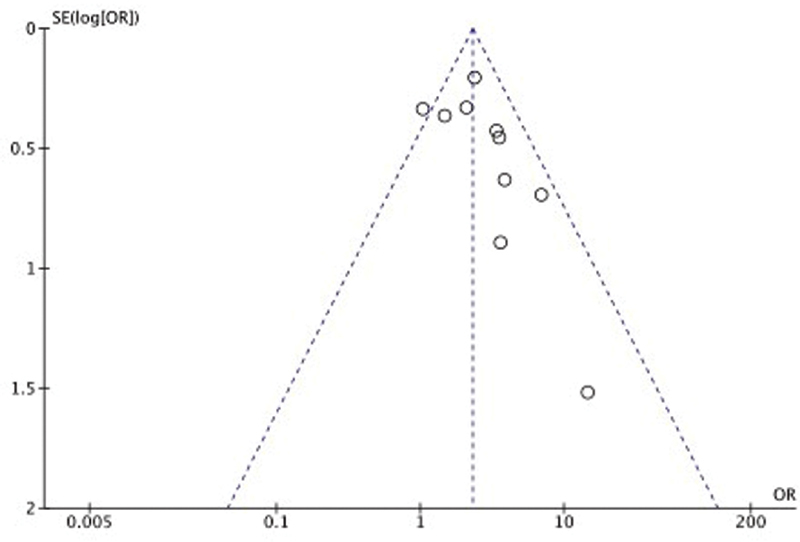
Funnel plot of the included studies in which abnormal Lp(a) was defined by values above a prespecified upper normal limit.

**Fig. 4:**
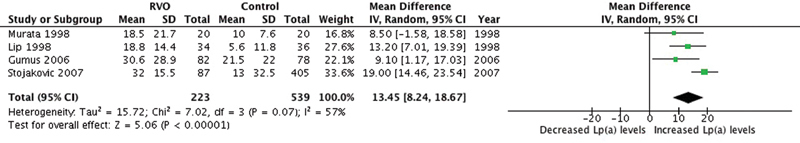
Forest plot evaluating the WMD in Lp(a) levels between patients with RVO and controls. CI, confidence interval. Lp(a), lipoprotein (a); RVO, retinal vein occlusion; WMD, weighted mean difference.

Out of 623 articles initially retrieved by our search strategy (35 Scopus, 556 Google Scholar, 32 PubMed), 610 were excluded because of reviews or case reports, studies not reporting Lp(a) plasma levels or studies in patients affected by retinal arterial thrombosis ( Fig. 1 ). At the end 13 studies, for a total number of 1,040 cases and 16,648 controls, were included in the analysis. 22 23 24 25 26 27 28 29 30 31 32 33 34 The characteristics of the included studies are summarized in Table 1 . Quality assessment of the studies was performed according to the criteria suggested by the Newcastle-Ottawa scale 38 ( Table 2 , Supplementary Table S1 ). Despite the nonprospective nature of the included studies, with only observational and in most cases cross-sectional studies, the overall quality was considered satisfactory being high or intermediate for all but one paper. Association between RVO risk and Lp(a) values was assessed in 10 studies 22 23 24 25 27 28 29 30 31 32 for a total of 837 cases and 16,129 controls. For the study by Kuhli-Hattenbach et al published in 2017 22 only patients aged >60 years were considered to avoid possible duplicates with results of a second study from the same authors published later. 28 Two studies by Glueck 31 32 were included because the prospective nature of the second one 32 contrarily to the observational nature of the first one excluded the possibility of duplicate cases. The Lp(a) plasma level cut-off values used for our analysis were chosen according to the normality ranges reported in 10 of the included studies (upper limit 35 mg/dL in two, 30 mg/dL in 6, 20 mg/dL in one, and 10 mg/dL in one). Prevalence of RVO was significantly higher in subjects with Lp(a) above upper limits compared with subjects within normal range (OR 2.38, 95% CI 1.7–3.34) ( Fig. 2 ). Heterogeneity among studies was not significant ( *I* ^2^ = 35%; Chi-square = 13.93, *p* = 0.12) ( Fig. 3 ). The presence of publication bias suggested by the asymmetry of funnel plot was confirmed by the Peters test 39 ( Supplementary Fig. S1 ). Lp(a)-attributable OVR risk-fraction was estimated to be 44%. Mean or median values of Lp(a) in subjects with RVO versus controls were reported in four studies 26 33 34 for a total of 223 cases and 539 controls. Lp(a) levels were significantly higher in patients with RVO than in controls (WMD 13.4 mg/dL, 95% CI 8.2–18.6). Heterogeneity among studies was significant ( *I* ^2^ = 57%, Chi-square= 7, *p* = 0.07) ( Fig. 4 ).

**Table 1:** Characteristics of the studies included in the analysis

Study name (ref)	RVO diagnosis	RVO site	Controls description	Patient description	RVO relevant characteristics compared with controls	Design	Sample size	Lp(a) measurement method	Lp(a) Cut-off a
Müller et al 1992 30	Not specified	Not specified	Healthy subjects	Adults with RVO	Same levels of cholesterol, triglycerides and LDL	Cross-sectional	84 cases 86 controls	Radial immunodiffusion or zone immunoelectrophoresis	Cut off (>30 mg/dL)
Bandello et al 1994 23	Fundus examination, angiography	CRVO	Healthy age-sex matched subjects	Adults with RVO	Higher D-dimer levels	Cross-sectional	40 cases 40 controls	ELISA	Cut off (>30 mg/dL)
Lip et al 1998 34	Clinical evaluation, fundus examination and angiography	CRVO, BRVO	Healthy age-sex matched subjects without AF.	Patients with RVO in sinus rhythm.	Higher prevalence of hypertension	Prospective	34 cases 36 controls	Immunoturbidimetry	Median
Murata et al 1998 24	Not reported	CRVO	Healthy subjects with cataract.	Adults with RVO	No further description	Prospective	20 cases 20 controls	Not reported	Cut off (>30 mg/dL)
Ribeaudeau-Saindelle et al 1998 29	Angiography	Not specified	Healthy age-sex and cardiovascular risk-matched subjects.	Adults with RVO	No differences in cardiovascular risk factors	Cross-sectionaI	132 cases 52 controls	Immunonephelometry	Cut-off (>10 mg/dL)
Glueck et al 1999 31	Fundus examination	Not specified	Healthy subjects	Adults with RVO	Higher prevalence of FV Leiden and lupus anticoagulant	Cross-sectionaI	16 cases 40 controls	Immunoassay	Cut-off (>35 mg/dL)
Wong et al 2005 27	Retinal photography	CRVO, BRVO	Age-matched subjects from ARIC study.	Subjects from ARIC study with RVO.	Higher prevalence of hypertension	Cross-sectionaI	34 cases 15,432 controls	ELISA	Cut-off (>20 mg/dL)
Gumus et al 2006 33	Complete ophthalmic evaluation	CRVO, BRVO	Healthy age-sex matched subjects.	Adults with RVO	Higher prevalence of hypertension, hyperhomocysteinemia and factor	Cross-sectional	82 cases 78 controls	Nephelometry	Above mean
Stojakovic et al 2007 26	Fundus examination	CRVO, BRVO	Healthy age-sex matched subjects.	Adults with RVO	Higher prevalence of hypertension	Retrospective	87 cases 405 controls	Immunoturbidimetry	Median
Sofi et al 2010 25	Fundus examination	Not specified	Healthy age-sex matched subjects.	Adults with RVO	Higher prevalence of hypertension, smoking, diabetes	Cross-sectionaI	262 cases 262 controls	Sandwich immunoassay	Cut-off (>30 mg/dL)
Glueck et al 2012 32	Fundus examination	CRVO	Healthy subjects	Adults with RVO	Higher prevalence of hyper homocysteinemia FVlll, anti-cardiolipin antibodies	Prospective	123 cases 102 controls	Immunoassay	Cut-off (>35 mg/dL)
Kuli-Hattenbach et al 2017 22	Best-corrected visual acuity, Intraocular pressure slit lamp examination.	CRVO, BRVO, HRVO	Healthy age-matched subjects with no history of VTE.	Adults with RVO	Higher prevalence of thrombophilia	Retrospective	20 cases 19 controls	Photometric sandwich enzyme immunoassay	Cut off (>30 mg/dL)
Kuli-Hattenbach et al 2018 28	Best-corrected visual acuity, intraocular pressure and anterior segment slit lamp examination.	CRVO, BRVO, HRVO	Healthy age-matched subjects with no history of VTE.	Adults with RVO	No further description	Retrospective	106 cases 76 controls	Photometric sandwich enzyme immunoassay	Cut off (>30 mg/dL)

Abbreviations: AF, atrial fibrillation; BRVO, branch retinal vein occlusion; CRVO, central retinal vein occlusion; ELISA, enzyme-linked immunosorbent assay; HRVO, hemiretinal vein occlusion; LDL, low density lipoprotein; VTE, venous thromboembolism.

a Cut-off values were all expressed in mg/dL.

**Table 2:** Quality assessment of the studies included in the analysis

Study name	Language	Year	New Ottawa Scale
Müller et al 30	English	1992	Intermediate
Bandello et al 23	English	1994	High
Lip et al 34	English	1998	Low
Murata et al 24	English	1998	Intermediate
Ribeaudeau-Saindelle et al 29	French	1998	Intermediate
Glueck et al 31	English	1999	High
Wong et al 27	English	2005	High
Gumus et al 33	English	2006	High
Stojakovic et al 26	English	2007	High
Sofi et al 25	English	2010	High
Glueck et al 32	English	2012	Low
KuIi-Hattenbach et al 22	English	2017	High
KuIi-Hattenbach et al 28	German	2018	Low

## Discussion

RVO is an unusual site vein thrombosis associated with potentially serious adverse outcomes, including blindness. In terms of predisposing factors RVO is closer to arterial rather than venous thrombosis because cardiovascular risk factors, instead of thrombophilia, seem to play a pre-eminent role. Our study, reporting the first systematic review and meta-analysis of investigations on the relationship between Lp(a) levels and RVO, supports the hypothesis that high Lp(a) is associated with RVO. 28 29 30 31 In fact Lp(a) levels above upper normal limits associated with RVO and patients with RVO had significantly higher plasma Lp(a) levels than controls.

The pathophysiologic role of Lp(a) in RVO may be explained by its multiple interactions with vascular and hemostatic homeostasis. Indeed, RVO is to a large extent the consequence of venous stasis provoked by compression from the near atherosclerotic arteriolar wall. 40 Therefore, differently from other risk factors, such as thrombophilia or hypercholesterolemia which act rather selectively on one of the two vascular beds, Lp(a) may favor RVO acting on both retinal arterioles and veins by enhancing vascular inflammation and by impairing fibrinolysis, thus favoring thrombosis. This twofold pathogenic activity of Lp(a) may be especially relevant in a condition like RVO which is on the border between venous and arterial thrombosis. 1 2 3 4 5

Based on our results, lowering Lp(a) represents an attractive approach to the prevention of RVO or its recurrence. Currently, the only recommended strategy to reduce Lp(a) remains plasma apheresis (expected reduction 60–80%) as there are no drugs able to selectively reduce Lp(a) levels, although some reduction has been reported with aspirin (15–20%), lomitapide and mipomersen (30%), PCSK9-inhibitors (30%), and nicotinic acid (38%). 10 However, in the near future lipid nanoparticle-vehiculated short interfering RNAs, such as antisense antiapo(a)oligonucleotides, are expected to revolutionize Lp(a) lowering therapy. 41 Indeed, in a recent phase II randomized placebo-controlled trial, the subcutaneous administration of the antisense oligonucleotide anti apo(a) AKCEA-APO(a)-Lrx significantly and dose dependently lowered Lp(a) plasma levels, with a maximum reduction of 80% at 6 months. 42 43 If this approach will obtain approval for clinical use, then its testing in patients with a previous RVO to prevent recurrence or in patients at high risk of RVO to prevent its occurrence will deserve to be assessed.

Our meta-analysis has some limitations. The first is the observational nature of all the included studies which, compared with randomized trials, may make the calculation of a single summary estimate of effect of exposure, in this case high Lp(a) levels, misleading. 44 45 However, the use of random effects model reduced this risk taking into account the possible variance among studies. 45 In addition, it is well established that Lp(a) levels may be influenced by several conditions, such as smoking and diabetes, which may act as confounding factors 9 and cardiovascular risk-matched selection of control was performed just in one of the studies included, thus limiting the possibility of considering these factors in the analysis. Moreover, we detected the likely presence of publication bias. Nevertheless, this was attenuated by the inclusion in our systematic review of gray literature and not published evidence in commercial publications. 46 Furthermore, a subgroup analysis according to site of occlusion (BRVO vs. CRVO) was not performed because separate information for these two types of RVO were not provided in the included studies, therefore a possible differential influence of Lp(a) on BRVO versus CRVO could not be excluded. In fact, some previous studies exploring the impact of other cardiovascular risk factors on RVO have shown that hypertension, peripheral arterial disease, diabetes mellitus, and atherosclerosis are significantly more associated with BRVO than with CRVO. 47 48 Therefore, further studies addressing the role of Lp(a) specifically in BRVO versus CRVO are highly warranted. HRVO, which is considered a third entity by some authors, 49 was reported only in one study 22 and may thus be underrepresented. Additionally, the cut-off values and laboratory methods used for the measurement of Lp(a) varied widely among the included studies and this may have affected the strength of the association between Lp(a) and RVO. This bias will be overcome only with the standardization of the measurement methods. Finally, despite the non-negligible number of included studies, the number of enrolled patients in our analysis was not large, however, still remarkable representing the largest collection of RVO cases related to Lp(a) levels reported so far.

## Conclusion

RVO remains an incompletely understood thrombotic disorder with many unsolved questions. To date, no obviously effective treatment is available, and several patients still develop blindness or severe visual impairment. Our data suggest that Lp(a) may represent an important factor in the pathogenesis of RVO and should be included among parameters to assess when evaluating the risk of RVO or RVO recurrence. Future prospective studies aimed to evaluate the role of Lp(a) in RVO risk and recurrence and the effect of Lp(a)-lowering treatments in patients with RVO is highly warranted.

## Notes

**What is Known about This Topic?**

- Retinal vein occlusion (RVO) pathogenesis is still unclear.
- Increased lipoprotein (a) [Lp(a)] has been associated with both arterial and venous thromboembolism.
- Some studies have shown higher Lp(a) levels in patients with RVO.

**What This Paper Add?**

- Systematic review and meta-analysis analyzing available evidence on the role of Lp(a) in RVO.
- Increased Lp(a) is associated with RVO incidence.
- Lp(a) levels are higher in RVO than non RVO subjects.
- Hypothesis-generating information for future clinical trials evaluating Lp(a) lowering drugs in RVO.
