# Supplementary material for: Role of Increased Lipoprotein (a) in Retinal Vein Occlusion: A Systematic Review and Meta-analysis
Source: TH Open. 2021 Jul 6;5(3):e295–302. doi: 10.1055/s-0041-1732803 (PMC8260280; doi:10.1055/s-0041-1732803)
Supplement: Supplementary file 1 — Supplementary Material [file 10-1055-s-0041-1732803-s210014.pdf]

Supplementary Table S1 Quality assessment of the included studies

	Muller et al[30]	Bandello et al[23]	Lip et al[34]	Murata et al[24]	Ribeaudeau-Saindelle et al[29]	Glueck et al[31]	Wong et al[27]	Gumus et al[33]	Stojakovic et al[26]	Sofi et al[25]	Glueck et al[32]	Kuli-Hattenbach et al[22]	Kuli-Hattenbach et al[28]
Total score (points)	6	7	6	5	7	6	7	7	7	7	5	7	5
Selection (maximum five stars)	****	***	***	***	***	***	***	***	***	***	***	***	***
Representativeness of the sample:													
Truly representative of the average in the target population[a]													
Na description of the sampling strategy													
Sample size:													
Justified and satisfactory[a]													
Not justified													
Ascertainment of the exposure (risk factor)													
Validated measurement tool[b]													
Nanvalidated measurement tool, but the tool is available or described[a]													
No description of the measurement tool													
Comparability (maximum 2 stars)	*	**	**	*	**	*	**	**	**	**	*	**	*
a. The study controls for the most important factor (select one)[a]													
b. The study control for any additional factor[a]													
Outcome (maximum three stars)	*	**	*	*	**	*	**	**	**	**	*	**	*
Assessment of the outcome													
a. Independent blind assessment[b]													
b. Record linkage[b]													
d. No description													
c. Self report[a]													
Statistical test													
The statistical test used to analyze the data are clearly described and appropriate, and the measurement of the association is presented, including confidence intervals and the probability level (*p*-value)[a]													
The statistical test is not appropriate, not described or incomplete													

Note: Low quality: 0–5 points; intermediate quality: 6 points; high quality: ≥7 points.
[a]Means that 1 point is assigned to the parameter.
[b]Means that 2 points are assigned to the parameter.

 © 2021. The Author(s).

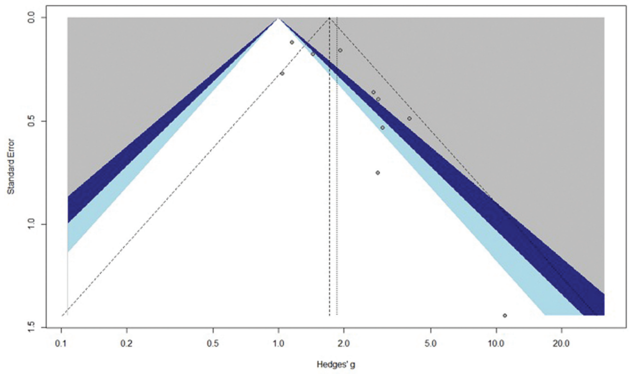

Supplementary Fig. S1 Publication bias visualization: Funnel plot (legend. *Blue* $p < 0.01$, *light blue* $p < 0.5$). Significant publication bias emerged with the Peters (sample estimate bias [se] =18.2, slope =0.15, *p*-value = 0.004188) and Egger's test (*p*-value = 0.02, se = 0.66).

 © 2021. The Author(s).
